# Non-Gaussian models of diffusion weighted imaging for detection and characterization of prostate cancer: a systematic review and meta-analysis

**DOI:** 10.1038/s41598-019-53350-8

**Published:** 2019-11-14

**Authors:** V. Brancato, C. Cavaliere, M. Salvatore, S. Monti

**Affiliations:** IRCCS SDN, Napoli, Italy

**Keywords:** Cancer imaging, Prostate

## Abstract

The importance of Diffusion Weighted Imaging (DWI) in prostate cancer (PCa) diagnosis have been widely handled in literature. In the last decade, due to the mono-exponential model limitations, several studies investigated non-Gaussian DWI models and their utility in PCa diagnosis. Since their results were often inconsistent and conflicting, we performed a systematic review of studies from 2012 examining the most commonly used Non-Gaussian DWI models for PCa detection and characterization. A meta-analysis was conducted to assess the ability of each Non-Gaussian model to detect PCa lesions and distinguish between low and intermediate/high grade lesions. Weighted mean differences and 95% confidence intervals were calculated and the heterogeneity was estimated using the I^2^ statistic. 29 studies were selected for the systematic review, whose results showed inconsistence and an unclear idea about the actual usefulness and the added value of the Non-Gaussian model parameters. 12 studies were considered in the meta-analyses, which showed statistical significance for several non-Gaussian parameters for PCa detection, and to a lesser extent for PCa characterization. Our findings showed that Non-Gaussian model parameters may potentially play a role in the detection and characterization of PCa but further studies are required to identify a standardized DWI acquisition protocol for PCa diagnosis.

## Introduction

Prostate cancer (PCa) is the second most common cancer among men^1^. Accurate detection and assessment of cancerous lesion aggressiveness according to the Gleason grading system^2^ is important for the most appropriate treatment strategy^3^. Currently, the most commonly used prostate cancer screening paradigm consists of the serum prostate-specific antigen (PSA) test, digital rectal examination, transrectal ultrasound (TRUS), and prostatic biopsies.

However, since these methods are often inaccurate and also invasive, there is a growing need for non-invasive tools to improve diagnosis of prostate cancer in terms of both detection and characterization.

Recently, a multiparametric Magnetic Resonance Imaging (MRI) based approach combining anatomic T1 or T2-weighted imaging with functional methods as Diffusion Weighted Imaging (DWI) and Dynamic Contrast Enhanced (DCE) imaging has significantly strengthened the role of MRI in diagnosis of PCa. There are several studies^4–6^ showing that multiparametric MRI is a useful tool that helps to detect prostate cancer foci, especially in patients with prior negative biopsy and permanently high PSA values. The classification used for evaluating the prostate as seen in MRI is known as PI-RADS (Prostate Imaging Reporting and Data System)^7^. This classification is based on a score from 1 to 5, with 1 indicating most probably benign lesions and 5 indicating lesions with a very high probability of malignancy. The use of PI-RADS, however, requires radiologists with high level of experience in MRI.

DWI technique exploits the diffusion phenomenon, which depends on the microscopic mobility of water molecules. Depending on how much water molecules movement is limited by tissue structure, the DWI signal intensity changes, and this has been proven useful in PCa to distinguish benign from malignant lesions and to characterize aggressiveness in terms of distinction between high- and low-grade tumors and correlation of tumor with Gleason Score (GS)^8–14^. Not only correlation with GS is important but it is of clinical importance to separate low Gleason grade PCa lesions from intermediate and high Gleason grade lesions^15^.

**Figure 1:**
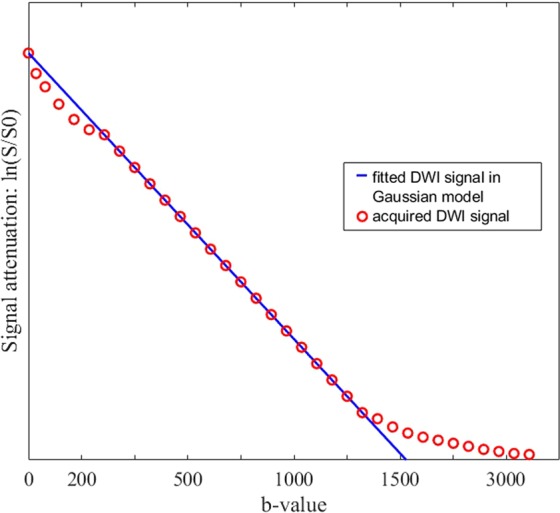
Example of fitted curve of diffusion weighted imaging (DWI) signal against the b-values. According to Gaussian model, when plotted against the b-values, the diffusion magnetic resonance (MR) signal ($\mathrm{ln}({\boldsymbol{S}}/{{\boldsymbol{S}}}_{0})$) would follow a straight line whose slope is the ADC (apparent diffusion coefficient). Non-Gaussian DWI models were introduced to describe the deviation of measured data from this expected line. Abbreviations: DWI, diffusion weighted imaging.

Several diffusion models have been studied in the field of PCa. Gaussian model has been largely used for cancer detection and characterization and has allowed meaningful results to be achieved. ADC values of PCa are generally lower than those of prostatic Normal Tissue (NT)^8,16^ and also reflect tumor aggressiveness, showing a negative correlation with GS^9,17^. However, the assumption at the basis of conventional DWI are not always accurate: water molecules experience vastly different environments in tissues, so *in vivo* water diffusion is much more complicated and often presents Non-Gaussian behavior. In terms of signal intensity, at low *b*-values (≤200 s/mm²) the signal attenuation is greater than expected, while it is lower at larger *b*-values (≥1500 s/mm²) (Fig. 1). Consequently, Non-Gaussian diffusion models have been proposed to better describe diffusion signal behavior, which can be directly related to tissue physiologic and pathologic characteristic. The most commonly used models are the Intravoxel Incoherent Motion (IVIM), the Diffusion Kurtosis Imaging (DKI), the Biexponential (BE), and the Stretched Exponential (SE).

There is a significant amount of studies aiming to investigate diagnostic performances of Non-Gaussian models, but their results suffer from inconsistency, insignificance and a not clear physical interpretation of Non-Gaussian parameters.

On this basis, the aim of our review is to evaluate the diagnostic performance of Non-Gaussian DWI models in terms of detection and characterization of prostate cancer (PCa). Specifically, after a detailed assessment, selection and systematic review of studies examining both Non-Gaussian and standard Gaussian DWI models, which will be shortly introduced in the following subsection, published data related to the most commonly used models parameters were statistically analyzed to evaluate their ability to detect PCa lesions and distinguish between low grade lesions (GS ≤ 6) and intermediate/high grade lesions (GS ≥ 7).

## Diffusion Weighted MRI Mathematical Models in PCa

The most commonly used mathematical models used to fit DWI signal are listed below:

1. Mono-exponential Model (ME)^18^
  $$\frac{S(b)}{{S}_{0}}=\exp (\,-\,b\cdot ADC)$$
2. Intravoxel Incoherent Motion Model (IVIM)^19^
  $$\frac{S(b)}{{S}_{0}}=(1-f)\cdot exp(-b\cdot D)+f\cdot exp(\,-\,b\cdot ({D}^{\ast }+D))$$
3. Biexponential Model (BE)^20–22^
  $$\frac{S(b)}{{S}_{0}}={f}_{slow}\cdot exp(-b\cdot {D}_{slow})+{f}_{fast}\cdot exp(\,-\,b\cdot {D}_{fast})$$
4. Stretched Exponential model (SE)^23^
  $$\frac{S(b)}{{S}_{0}}=\exp [\,-\,{(b\cdot DDC)}^{\alpha }]$$
5. Diffusion Kurtosis Imaging (DKI)^24^

$$\frac{S(b)}{{S}_{0}}=exp(-b\cdot {D}_{K}+\frac{{b}^{2}\cdot {{D}_{K}}^{2}\cdot K}{6})$$

In all the equations, *S(b)* is the measured signal intensity at a certain *b*, *S*_0_ the signal intensity at *b* = 0 and *b* is a factor that measures the degree of diffusion weighting applied. In the ME model, *i.e*. the standard gaussian model, *ADC* is the Apparent Diffusion Coefficient, an average value related to diffusion. In the IVIM model f is the perfusion fraction, D the molecular diffusion coefficient and D* the pseudo-diffusion coefficient. ${D}_{fast}$ and ${D}_{slow}$ are, respectively, fast and slow diffusion coefficients of the BE model and ${f}_{fast}$ and ${f}_{slow}$ their amplitudes. DDC is the distributed diffusion coefficient of the SE model, and α is the heterogeneity index. Finally, in the DKI model, D_K_ is the diffusion coefficient corrected for kurtosis, and K is the kurtosis coefficient. See Supplementary Material-S1- for more details on Non-Gaussian DWI models.

## Methods

### Search strategy and selection criteria

A systematic search for relevant published studies examining and, if any, comparing mono-exponential DWI and Non-Gaussian DWI models in PCa diagnosis was conducted. The most relevant scientific electronic databases (PubMed, Cochrane Library, MEDLINE, ScienceDirect, Google Scholar) were comprehensively explored and used to build the search. Only studies published after 2012 were selected. The search strategy included the key terms listed in Supplementary materials -S2-. The literature search was restricted to English language publications and studies of human subject.

Two reviewers, after having independently screened identified titles and abstracts, assessed the full text of the articles that evaluated mono-exponential DWI and at least one Non-Gaussian model, between IVIM, BE, SE, DKI, for diagnosis of PCa (detection and/or characterization of aggressiveness) and were not review articles. For articles meeting these criteria with full text available, the following further selection criteria had to be fulfilled: presence of PCa histopathological confirmation (either from biopsy or radical prostatectomy), of information about DW-MRI protocol, of a maximum b-value at least equal to 1500 s/mm² (if DKI was performed). Moreover, articles were excluded if computed b-values were used, if they concerned only model fitting quality or differentiation between PCa and benign lesions, not evaluating NT. The above-mentioned selection procedure was employed to perform a systematic review, providing a qualitative synthesis of currently used Non-Gaussian DWI models in PCa diagnosis.

In order to perform a meta-analysis, a further exclusion procedure was performed: studies were removed if they focused only on correlation of parameters with GS, and not on their ability to differentiate low-GS from high-GS tumors; if number of analyzed region of interest (ROI) was not mentioned; if data were reported in the form of mean and Inter Quartile Range (IQR), median and IQR, or median and 95% Confidence Interval (CI); if mean and standard deviation of parameters were not reported (or could not be calculated); if *b* = 0 mm^2^/s was not included in the DWI protocol. Moreover, if there was a high heterogeneity in fitting functions/procedures used for biexponential models, statistical analyses had not been conducted.

### Planning of the study

The articles were classified according to the Non-Gaussian models examined and the diagnostic purpose they had, as reported in Table 1.

**Table 1:** Article classifications according to the Non-Gaussian model examined and the diagnostic purpose of the study. Abbreviations: PCa, prostate cancer.

Classification based on Non-Gaussianmodels	Classification based on PCa diagnosis
Intravoxel Incoherent Motion Model (**IVIM**)	PCa detection
Biexponential Model (**BE**)	Characterization of PCa aggressiveness
Stretched Exponential Model (**SE**)	
Diffusion Kurtosis Imaging (**DKI**)	

**Figure 2:**
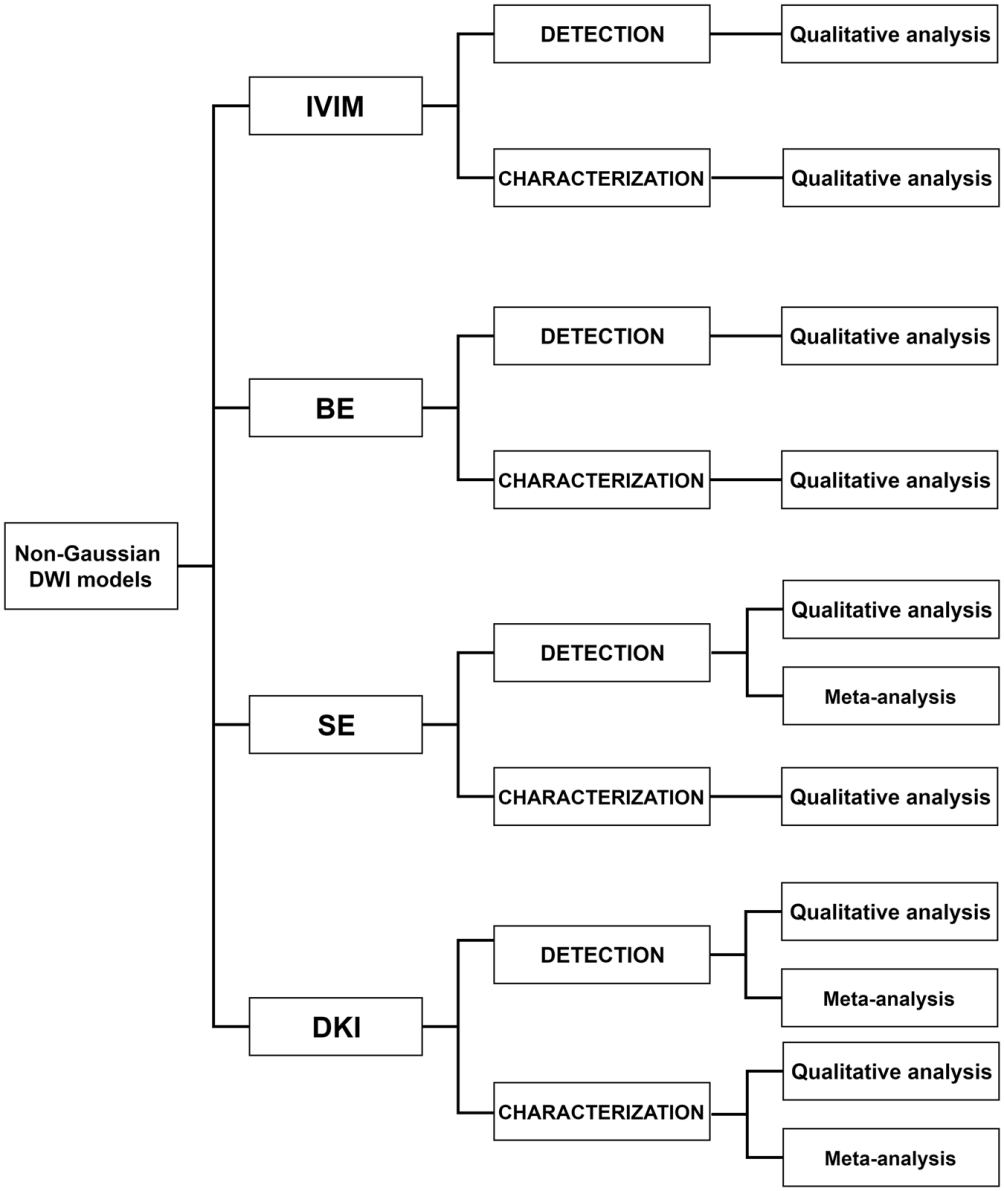
Scheme reporting planning of the study. Abbreviations: DWI, Diffusion Weighted Imaging; IVIM, Intravoxel Incoherent Motion model; BE, Biexponential model; SE, Stretched Exponential model; DKI, Diffusion Kurtosis Imaging.

Consequently, our work was organized in accordance with Fig. 2: for each Non-Gaussian model, a qualitative analysis followed by a meta-analysis (when a sufficient number of articles was available after the application of selection criteria) was performed both to assess the difference of the mean value of IVIM, BE, DKI and SE parameters between NT and PCa (PCa detection) and to assess the parameters mean differences between low GS and intermediate/high GS PCa (characterization of PCa aggressiveness).

### Meta-analyses methods

Meta-analyses were conducted in accordance with the Preferred Reporting Items for Systematic Reviews and Meta-Analyses (PRISMA) statement^25^ (See Supplementary Materials-S3-for PRISMA Checklist).

Although there were not enough data for performing an assessment of diagnostic accuracy, the quality of studies included in meta-analysis was evaluated, using the QUADAS-2^26^ tool included in RevMan (version 5.3, The Cochrane Collaboration). The quality of each study was evaluated by two reviewers independently and any disagreement was resolved by consensus. If meta-analysis included a number of studies superior to 10^27,28^, publication bias was assessed by visually inspecting a funnel plot.

Mean and Standard Deviation (SD) of diffusion model parameters were extracted from each selected article. To analyze the differences between groups, Weighted Mean Differences (WMD) and 95% CI were calculated. The overall result was considered statistically significant if the test for overall effect returns a probability value lower than 0.05.

Heterogeneity among included studies was assessed using the Q statistic of the chi-square value test and the inconsistency index of Higgings^29^ I^2^, with values of 25%, 50%, and 75% considered as low, moderate, and high, respectively. If the P-value of heterogeneity test was less than 0.1 or the I^2^-value was greater than 50%, the summary estimate was analyzed by a random-effects model^30^. Otherwise, a fixed-effects model was applied.

All statistical computations were performed using RevMan (version 5.3, The Cochrane Collaboration).

## Results

**Figure 3:**
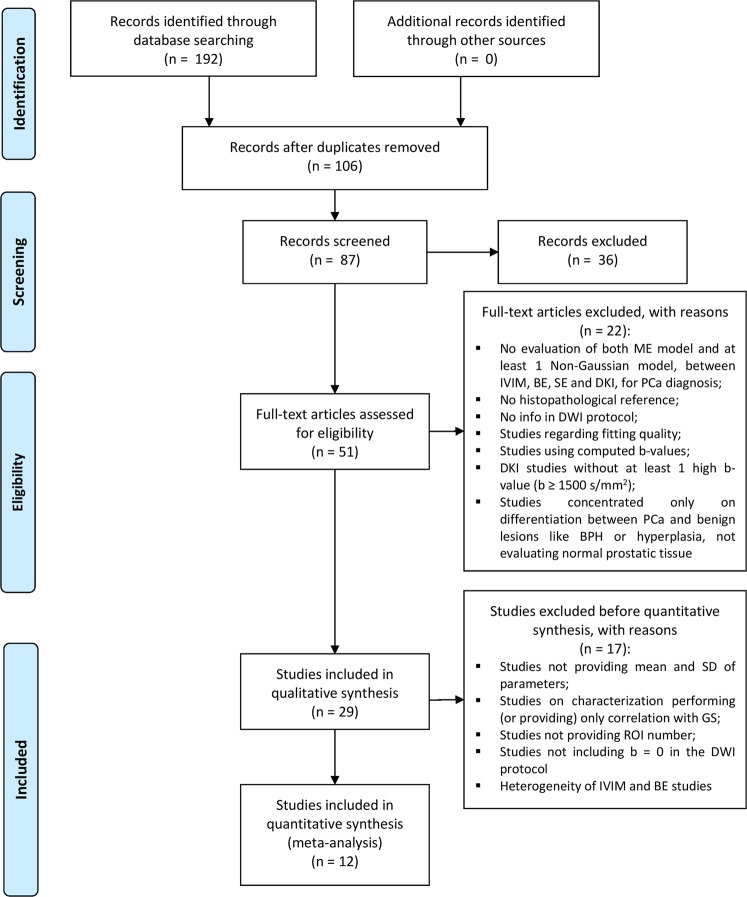
PRISMA flow diagram of the study selection procedure. Abbreviations: DWI, Diffusion Weighted Imaging; IVIM, Intravoxel Incoherent Motion model; BE, Biexponential model; SE, Stretched Exponential model; DKI, Diffusion Kurtosis Imaging; BPH, benign prostatic hypertropia; SD, standard deviation; GS, Gleason Score; ROI, region of interest.

The PRISMA flow diagram of included studies according to the inclusion and exclusion criteria is reported in Fig. 3.

### Results of qualitative analysis

29 studies fulfill all the inclusion criteria and were involved in the qualitative analysis: their characteristics are summarized in Tables 2 and 3.

**Table 2:** Selected studies for qualitative and quantitative analysis.

Author	Year	Non-Gaussian Models	Purpose (D/C)	Sd	No. of PCa patients	No. of PCa regions	No. of NT regions	No. of Low GS regions (<=6)	No. of High GS regions (>6)	Included in MA	Reasons for exclusion from MA
Shinmoto *et al*.^31^	2012	IVIM	D	R	26	N/E	N/E	—	—	No	Study on IVIM model
Liu *et al*.^45^	2013	BE	D	P	23	N/E	N/E	—	—	No	Study on BE model
Kuru *et al*.^41^	2014	IVIM	D, C	R	27	N/E	N/E	N/E	N/E	No	Study on IVIM model
Zhang *et al*.^37^	2015	IVIM	C	R	48	—	—	N/E	N/E	No	Study on IVIM model
Martin *et al*.^32^	2014	IVIM	D	R	36	N/E	N/E	—	—	No	Study on IVIM model
Valerio *et al*.^42^	2016	IVIM	D, C	P	53	N/E	N/E	N/E	N/E	No	Study on IVIM model
Yang *et al*.^38^	2016	IVIM	C	R	41	—	—	N/E	N/E	No	Study on IVIM model
Barbieri *et al*.^39^	2017	IVIM	C	P	84	—	—	N/E	N/E	No	Only analysis of correlation with GS reported
Bao *et al*.^40^	2017	IVIM	C	P	30	—	—	N/E	N/E	No	Only analysis of correlation with GS reported
Pesapane *et al*.^43^	2017	IVIM	D, C	P	31	N/E	N/E	N/E	N/E	No	Study on IVIM model
Liu *et al*.^49^	2015	SE	D	P	27	31	62	—	—	Yes	
Liu *et al*.^50^	2018	SE	C	R	75	—	—	N/E	N/E	Yes	
Rosenkrantz *et al*.^54^	2012	DKI	D, C	R	47	121	47	51	70	Yes	
Tamura *et al*.^51^	2014	DKI	D	R	20	24	20	—	—	Yes	
Suo *et al*.^55^	2014	DKI	D, C	R	19	19	19	9	10	Yes	
Roethke *et al*.^56^	2015	DKI	D, C	R	55	55	55	12	43	Yes	
Wang *et al*.^52^	2015	DKI	C	R	110	—	—	49	77	Yes	
Tamada *et al*.^57^	2017	DKI	D, C	R	285	285	285	73	311	Yes	
Wang *et al*.^53^	2018	DKI	C	R	67	—	—	N/E	N/E	No	b = 0 not included in DWI protocol
Quentin *et al*.^33^	2012	IVIM	D	P	8	N/E	N/E	—	—	No	Study on IVIM model
Mazzoni *et al*.^34^	2014	IVIM, DKI	D	R	57	73	45	—	—	Yes	Exclusion of IVIM model
Ueda *et al*.^35^	2016	IVIM	D	R	63	64	64	—	—	No	Study on IVIM model
Jambor *et al*.^46^	2015	BE, DKI, SE	D	P	16	N/E	N/E	—	—	No	Mean - SD values not reported
Toivonen *et al*.^48^	2015	BE, DKI, SE	D, C	P	50	N/E	N/E	N/E	N/E	No	Mean - SD values notreported
Feng *et al*.^36^	2017	IVIM, DKI, SE	D	P	56	138	198	—	—	Yes	Exclusion of IVIM model
Barrett *et al*.^58^	2017	DKI	D, C	P	30	N/E	N/E	N/E	N/E	No	Mean - SD values notreported
Merisaari *et al*.^44^	2017	IVIM, SE	D, C	P	81	N/E	N/E	N/E	N/E	No	Mean - SD values not reported
Mazaheri *et al*.^47^	2018	BE, DKI, SE	D	R	55	55	55	—	—	Yes	Exclusion of BE model
Langkilde *et al*.^15^	2018	BE, DKI, SE	D, C	R	40	40	111	N/A	N/A	Yes	Exclusion of BE model

Abbreviations: IVIM, Intravoxel Incoherent Motion Model; BE, Biexponential Model; DKI, Diffusion Kurtosis Imaging; SE, Stretched Exponential Model; D, detection; C, characterization; Sd, study design; P, prospective; R, retrospective; PCa, prostate cancer; NT, normal tissue; GS, Gleason Score; MA, meta-analysis; SD, standard deviation; N/E, not extracted because not necessary for quantitative analysis; —, non-existent. Notes: the number of PCa regions, Low GS regions and High GS regions were reported as sum of all PCa, Low GS or High GS regions respectively, regardless of the affected prostate zone (peripheral zone, transition zone, central gland); the number of NT regions were reported as sum of all NT regions, regardless of the affected prostate zone and the patient on which the ROI was placed (e.g. healthy volunteer, PCa patient). The column “Reason for exclusion from MA” reported the reason why the study under investigation, or which analyzed model, was excluded from the meta-analysis. As regards IVIM and BE models, they were excluded from meta-analysis due to heterogeneity in fitting functions/procedures among selected studies and this was indicated by the sentences “Study on IVIM model” or “Study on BE model”, if the study included only these models, “Exclusion of IVIM model” or “Exclusion of BE model”, if the study reported other Non-Gaussian DWI models that were, instead, retained into meta-analysis.

**Table 3:** Imaging characteristics.

Author	FS [T]	TR/TE [ms/ms]	Seq.	b-values [s/mm^2^]	Non-Gaussian fitting function(s)	Non-Gaussian Parameters	Fitting procedure	Initialization values	Methods to prevent finding local minima
Shinmoto *et al*.^31^	3.0	5132/40	NR	0, 10, 20, 30, 50, 80, 100, 200, 400, 1000	S(b)/S_0_ = (1-f)∙exp(−b∙D) + f∙exp(−b∙(D* + D))	D, D*, f	NR	NR	NR
Liu *et al*.^45^	3.0	4000/71.9	SS-EPI	For ADC: 0, 1000; For BE: 0, 300, 600, 900, 1200, 1500, 1800, 2100, 2400, 2700, 3000	S(b)/S_0_ = f_slow_∙exp(−b∙D_slow_) + f_fast_∙exp(−b∙D_fast_)	D_slow_, D_fast_, f	NR	NR	NR
Kuru *et al*.^41^	3.0	3100/52	SS-EPI	0, 50, 100, 150, 200, 250, 800	S(b)/S_0_ = (1-f)∙exp(−b∙D) + f∙exp(−b∙(D* + D))	M1: D, D*, f M2: D, f	M1: SLF for D and f, NLRF for D*; M2: BeF using LMa with D* fixed to 20 µm^2^/ms	NR	NR
Zhang *et al*.^37^	3.0	6000/72	SS-EPI	0, 50, 150, 300, 600, 900	S(b)/S_0_ = (1-f)∙exp(−b∙D) + f∙exp(−b∙D*)	D, D*, f	BeF using LMa	NR	NR
Martin *et al*.^32^	3.0	5000/54	SS-EPI	0, 20, 40, 100, 300, 500, 1000, 2000	S(b)/S_0_ = (1-f)∙exp(−b∙D) + f∙exp(−b∙(D* + D))	D, f	NR	NR	NR
Valerio *et al*.^42^	3.0	3100/102	NR	For ADC: 0, 500, 1000, 3000; For IVIM: 0, 10, 20, 30, 40, 50, 80, 100, 200, 400, 800	S(b)/S_0_ = (1-f)∙exp(−b∙D) + f∙exp(−b∙D*)	D, D*, f	NR	D: [0–10]∙10^−3^ mm^2^/s D*: [10–150]∙10^−3^ mm^2^/s f: [0–1]	NR
Yang *et al*.^38^	3.0	5000/90	SS-EPI	0, 10, 20, 50, 100, 200, 500, 800	S(b)/S_0_ = (1-f)∙exp(−b∙D) + f∙exp(−b∙D*)	D, D*, f	BeF using LMa	NR	NR
Barbieri *et al*.^39^	3.0	2600/58	SS-EPI	0, 10, 20, 50, 130, 270, 500, 900	S(b)/S_0_ = (1-f)∙exp(−b∙D) + f∙exp(−b∙D*)	D, D*, f	Bpb	NR	NR
Bao *et al*.^40^	3.0	6800/98	SS-EPI	0, 50, 100, 150, 200, 500, 1000	S(b)/S_0_ = (1-f)∙exp(−b∙D) + f∙exp(−b∙D*)	D, D*, f	NR	NR	NR
Pesapane *et al*.^43^	1.5	7000/10	SS-EPI	For ADC: 0, 1000, 2000; For IVIM: 0, 10, 20, 30, 50, 80, 100, 200, 400, 800	S(b)/S_0_ = (1-f)∙exp(−b∙D) + f∙exp(−b∙(D* + D))	D, D*, f	NR	NR	NR
Liu *et al*.^49^	3.0	4000/71.9	SS-EPI	0, 500, 1000, 2000	S(b)/S_0_ = exp[−(b∙DDC)^α^]	DDC, α	NR	—	NR
Liu *et al*.^50^	3.0	4000/71.9	SS-EPI	0, 500, 1000, 2000	S(b)/S_0_ = exp[−(b∙DDC)^α^]	DDC, α	NR	—	NR
Rosenkrantz *et al*.^54^	3.0	3500/81	SS-EPI	0, 500, 1000, 1500, 2000	S(b)/S_0_ = exp(−b∙D_K_ + b^2^∙D_K_^2^∙K/6)	D_K_, K	NR	—	NR
Tamura *et al*.^51^	3.0	5000/49	SS-EPI	0, 10, 20, 30, 50, 80, 100, 200, 400, 1000, 1500	S(b)/S_0_ = exp(−b∙D_K_ + b^2^∙D_K_^2^∙K/6)	D_K_, K	NR	—	NR
Suo *et al*.^55^	3.0	3940/106	SS-EPI	0, 500, 800, 1200, 1500, 2000	S(b)/S_0_ = exp(−b∙D_K_ + b^2^∙D_K_^2^∙K/6)	D_K_, K	NLLS	—	NR
Roethke *et al*.^56^	3.0	For ADC: 3100/52 For DKI: 2700/70	SS-EPI	For ADC: 0, 800 For DKI: 0, 50, 250, 500, 750, 1000, 1250, 1500, 2000	S(b)/S_0_ = exp(−b∙D_K_ + b^2^∙D_K_^2^∙K/6)	D_K_, K	LMa	—	NR
Wang *et al*.^52^	3.0	6800/98	SS-EPI	0, 700, 1400, 2100	S(b)/S_0_ = exp(−b∙D_K_ + b^2^∙D_K_^2^∙K/6)	D_K_, K	NR	—	NR
Tamada *et al*.^57^	3.0	3500/81	SS-EPI	For ADC: 0, 1000 For DKI: 0, 500, 1000, 1500, 2000”	S(b)/S_0_ = exp(−b∙D_K_ + b^2^∙D_K_^2^∙K/6)	K	NR	—	NR
Wang *et al*.^53^	3.0	4500/95	SS-EPI	200, 500, 1000, 1500, 2000	S(b)/S_0_ = exp(−b∙D_K_ + b^2^∙D_K_^2^∙K/6)	K	NR	—	NR
Quentin *et al*.^33^	3.0	2600/89	SS-EPI	0, 50, 100, 150, 200, 300, 400, 500, 600, 700, 800	S(b)/S_0_ = (1-f)∙exp(−b∙D) + f∙exp(−b∙(D* + D))	D, D*, f	NR	NR	NR
Mazzoni *et al*.^34^	3.0	2100/69	SS-EPI	0, 50, 100, 150, 200, 250, 400, 650, 800, 1000, 1400, 1800, 2300 Different ranges used: 0–2300 (group A); 0–1800 (group B); 0–800 (group C)	IVIM: S(b)/S_0_ = (1-f)∙exp(−b∙D) + f∙exp(−b∙D*) DKI: S(b)/S_0_ = exp(−b∙D_K_ + b^2^∙D_K_^2^∙K/6)	D, D*, f, D_K_, K	NR	NR	NR
Ueda *et al*.^35^	3.0	4000/65	SS-EPI	0, 50, 100, 200, 500, 1000, 2000, 3000	S(b)/S_0_ = (1-f)∙exp(−b∙D) + f∙exp(−b∙D*)	D, D*, f	SLF for D	NR	NR
Jambor *et al*.^46^	3.0	3141/51	SS-EPI	For HV: 0, 50, 100, 200, 350, 500, 650, 800, 950, 1100, 1250, 1400, 1550, 1700, 1850, 2000 For PCa patients: 0, 100, 300, 500, 700, 900, 1100, 1300, 1500, 1700, 1900, 2000	BE: S(b)/S_0_ = f_slow_∙exp(−b∙D_slow_) + f_fast_∙exp(−b∙D_fast_) DKI: S(b)/S_0_ = exp(−b∙D_K_ + b^2^∙D_K_^2^∙K/6)	D_slow_, D_fast_, f_fast_, D_K_, K, DDC, α	NR	For PCa: D_fast_:1.0–9.0 (ss 0.2) µm^2^/ms D_slow_:0.0–4.0 (ss 0.02) µm^2^/ms f:0.2–1.0 (ss 0.1) For HV: D_fast_:1.0–7.0 (ss 0.1) µm^2^/ms D_slow_:0.0–2.0 (ss 0.01) µm^2^/ms f:0.2–1.0 (ss 0.1)	Multiple initialization values
Toivonen *et al*.^48^	3.0	3141/51	SS-EPI	0, 100, 300, 500, 700, 900, 1100, 1300, 1500, 1700, 1900, 2000	BE: S(b)/S_0_ = f_slow_∙exp(−b∙D_slow_) + f_fast_∙exp(−b∙D_fast_) DKI: S(b)/S_0_ = exp(−b∙D_K_ + b^2^∙D_K_^2^∙K/6)	D_slow_, D_fast_, f_fast_, D_K_, K, DDC, α	BeF using LMa	D_fast_:1.0–9.0 (ss 0.2) µm^2^/ms D_slow_:0.0–4.0 (ss 0.02) µm^2^/ms f:0.2–1.0 (ss 0.1)	Multiple initialization values
Feng *et al*.^36^	3.0	2500/84.1	SS-EPI	0, 20, 50, 80, 100, 150, 200, 400, 600, 800, 1000, 1200, 1500, 1800, 2000, 2400, 2800, 3200, 3600, 4000, 4500 Different ranges used: 0–1000; 0–2000; 0–3200; 0–4500	DKI: S(b)/S_0_ = exp(−b∙D_K_ + b^2^∙D_K_^2^∙K/6) SE: S(b)/S_0_ = exp[−(b∙DDC)^α^]	D, D*, f, D_K_, K, DDC, α	BeF using LMa	NR	NR
Barrett *et al*.^58^	3.0	For DWI: 4000/70–75 For DKI: 6000/94	SS-EPI	For ADC: 0, 150, 1000, 1400; For DKI: 0, 150, 450, 800, 1150, 1500	S(b)/S_0_ = exp(−b∙D_K_ + b^2^∙D_K_^2^∙K/6)	D_K_, K	NR	—	NR
Merisaari *et al*.^44^	3.0	1394/44	SS-EPI	0, 2, 4, 6, 9, 12, 14, 18, 23, 28, 50, 100, 300, 500	IVIM: S(b)/S_0_ = (1-f)∙exp(−b∙D) + f∙exp(−b∙D*) SE: S(b)/S_0_ = exp[−(b∙DDC)^α^]	D, D*, f, DDC, α	For IVIM: NLLS, SM, OSM, NNLS, delta. For DKI and SE: LMa	NLLS: D:0.01–3.5 (ss 0.1) µm^2^/ms D*:0.1–28.0 (ss 1.0) µm^2^/ms f:0.001–0.25 (ss 0.01) SM, OSM: D:0.01–3.5 (ss 0.1) µm^2^/ms D*:0.1–25.0 (ss 1.0) µm^2^/ms f:0.001–0.25 (ss 0.01) NNLS: D:0.01–4.0 (ss 0.02) µm^2^/ms D*:1–9.0 (ss 0.2) µm^2^/ms f:0.0–1.0 delta: D:0.01–2.0 (ss 0.02) µm^2^/ms f:0.001–0.1	Multiple initialization values
Mazaheri *et al*.^47^	3.0	3000–4000/78.2–80.4	SS-EPI	0, 600, 800, 1000, 1200, 1400, 1800, 2000	BE: S(b)/S_0_ = f_slow_∙exp(−b∙D_slow_) + f_fast_∙exp(−b∙D_fast_) DKI: S(b)/S_0_ = exp(−b∙D_K_ + b^2^∙D_K_^2^∙K/6) SE: S(b)/S_0_ = exp[−(b∙DDC)^α^]	D_slow_, D_fast_, f_fast_, D_K_, K, DDC, α	BeF using LMa	NR	
Langkilde *et al*.^15^	3.0	4000/~100	NR	0, 250, 500, 750, 1000, 1250, 1500, 1750, 2000, 2250, 2500, 2750, 3000, 3250, 3500	BE: S(b)/S_0_ = f_slow_∙exp(−b∙D_slow_) + f_fast_∙exp(−b∙D_fast_) DKI: S(b)/S_0_ = exp(−b∙D_K_ + b^2^∙D_K_^2^∙K/6) SE: S(b)/S_0_ = exp[−(b∙DDC)^α^]	D_slow_, D_fast_, f_fast_, D_K_, K, DDC, α	BeF using LMa	NR	

Abbreviations: IVIM, Intravoxel Incoherent Motion Model; BE, Biexponential Model; DKI, Diffusion Kurtosis Imaging; SE, Stretched Exponential Model D, molecular diffusion coefficient; D*, pseudo-diffusion coefficient; f, perfusion fraction; D_slow_, slow diffusion coefficient; D_fast_, fast diffusion coefficient; f_slow_, amplitude of slow diffusion coefficient; f_fast_, amplitude of fast diffusion coefficient; D_K_, diffusion coefficient corrected for kurtosis; K, kurtosis coefficient; DDC, distributed diffusion coefficient; α, heterogeneity index; ADC, apparent diffusion coefficient; FS, field strength; T, Tesla; TR, Repetition Time; TE, Echo Time; ms, milliseconds; Seq., diffusion sequence; HV, healthy volunteers; SS-EPI, Single-Shot Echo-Planar Imaging;BeF, Biexponential Fit; SLF, Simplified Linear Fit; NLRF, Non-Linear Regression Fit; LMa, Levemberg-Marquardt algorithm; Bpb, Bayesian probability-based approach; NNLS, Non Negative Least Square; SM, Segmented Method; OSM, Oversegmented Method; ss, step size; NR, not reported. Diffusion times column was not added because only 4 studies provided this acquisition parameter (see Supplementary Material –S6- for more details).

In all selected studies, ADC value proved to be a useful tool for discriminating both NT from PCa and low- from high-GS tumors, with lower values in tumors than in NT and in high-GS tumors than in low-GS tumors. On the other hand, findings on Non-Gaussian model features are not always equally consistent.

### Studies on IVIM

14 studies included IVIM comparison with ADC: 6 on detection^31–36^, 4on characterization^37–40^, and 4 on both^41–44^. Most studies on detection found that D was significantly smaller in PCa when compared to NTs^31–36,41–44^, but Mazzoni *et al*.^34^ revealed a dependence on b-value range, showing statistical significance only using a maximum b value of 800 s/mm^2^. Results on f, when significant, revealed a lower value in PCa than in NT^31–34,36,43^, with the exception of study by Ueda *et al*.^35^ who, conversely, found f significantly higher in peripheral zone (PZ) cancer than in normal PZ tissue, although they found no significance when performing the same analysis in transitional zone (TZ). Results on D* were found to be not significant in many studies^33–36^. Pesapane *et al*.^43^ found that D* was significantly higher in tumor tissue when compared to NT, in accordance with Kuru *et al*.^41^ and Valerio *et al*.^42^. While Linear Discriminant Analysis performed by Pesapane *et al*.^43^ and Valerio *et al*.^42^ showed that the additional use of IVIM increased performances of conventional T2/DWI for PCa detection, ROC analysis performed by Kuru *et al*. proved that none of IVIM parameters yielded a clear added value, such as in Feng *et al*.^36^. Whereas, Martin *et al*.^32^ found D and f to be statistically significant, obtaining also a slightly higher AUC for these two parameters than for ADC. In PCa aggressiveness characterization, D seems to be the most performing IVIM parameter^37–42^, resulting lower in high- than in low-GS tumors in accordance with ROC analysis performed by Yang *et al*.^38^. About D*, only Valerio *et al*.^42^ found its value to be significantly higher in high- than in low-GS PCa, while f was considered unable to discriminate between GS grade in all selected studies. Pesapane *et al*.^43^ identified all three IVIM parameters as not useful for characterization on PCa aggressiveness. In addition, some studies carried out a correlation analysis between the IVIM parameters and the GS: they revealed a significative negative correlation between D and GS^38–40^, while no correlation was found for f and D*. Merisaari *et al*.^44^ used machine learning techniques in order to evaluate if GS prediction could be improved using ADC in combination with IVIM, but they concluded that ADC continued to be the best-performing parameter for this scope.

### Studies on BE

5 studies included BE comparison with ADC:4 on detection^15,45–47^ and 1 on both detection and characterization^48^. Liu *et al*.^45^ found all three BE parameters significantly lower in PCa than in NT, in accordance with Mazaheri *et al*. and Langkilde *et al*.^15,47^, who found similar results for D_slow_ and f_fast_, but any significance for D_fast_. In contrast, Jambor *et al*.^46^ although identified in BE the best performance for normal and PCa data fitting, found median values of its three parameters not reaching statistical significance for PCa detection. Toivonen *et al*.^48^ evaluated BE model in terms of both detection and characterization, performing two ROC analysis and a correlation analysis with GS. Their results did not demonstrate improvements in PCa detection and characterization compared with mono-exponential model and only f showed a significant negative correlation with GS.

### Studies on SE

8 studies included SE comparison with ADC:5 on detection^15,36,46,47,49^, 2 on characterization^44,50^, and 1 on both^48^. According to Mazaheri *et al*.^47^ and Liu *et al*.^49^, DDC and αin PCa were significantly lower than in NT, showing that SE model parameters could provide additional information to ME model. These results are consistent with those of Feng *et al*.^36^ who, however, also performed a ROC analysis showing good performances for both parameters, but no significant performances improvement with respect to ADC. Jambor *et al*.^46^ found similar results for DDC, but not for α. Even ROC analysis performed by Toivonen *et al*.^48^ reveals significance for DDC, both in PCa detection and characterization, showing also a negative correlation between DDC and GS, even if performances are not such to substitute the ME model. In the study by Liu *et al*.^50^ DDC was found to be the only SE parameter able to differentiate low- from high-grade tumors, showing a significant negative correlation with GS. Merisaari *et al*.^44^ found a statistically significant correlation for both DDC and αwith GS (negative and positive respectively), a high AUC for low- versus high-GS classification and an AUC improvement combining DDC and α by means of machine learning. Nevertheless, none of these parameters or their combination considerably outperformed ADC parameter of ME model.

### Studies on DKI

15 studies included DKI comparison with ADC. Of these, 6 were on detection^34,36,46,47,51^, 2 on characterization^52,53^, and 7 dealt with both together^15,48,54–58^.

All the studies revealed a significantly higher K value in tumor tissue than in NT, with increasing trend in high GS lesions, but there were different opinions regarding the added value offered by the use of DKI for PCa diagnosis. Preliminary findings by Rosenkrantz *et al*.^54^ indicated higher capability of DKI model for both distinguishing benign from malignant PCa lesions and low- from high-grade PCa lesions when compared to ADC, by means of ROC analyses. They found K significantly higher and D_K_ significantly lower in PCa than in NT, and the same behavior was observed when low- and high- GS lesions were compared. The findings on PCa detection were successively confirmed by Tamura *et al*.^51^, Jambor *et al*.^46^, Suo *et al*.^55^, Mazaheri *et al*.^47^, and Mazzoni *et al*.^34^ who found that K was the best-performing parameter. Feng *et al*.^36^, Roethke *et al*.^56^, and Langkilde *et al*.^15^, although their statistical significance test results were in accordance with the above-mentioned studies, revealed not sufficiently higher performance of D_K_ or K compared to ADC at ROC analysis, in accordance with Toivonen *et al*.^48^. A similar conclusion was made my Tamada *et al*.^57^, who studied only K parameter. In the last 4 papers the authors, together with Wang *et al*.^53^, found similar results also in PCa characterization. Suo *et al*.^55^ found significant differences among low- and high-grade lesions for K value, but not for D_K_ value. Conversely, results by Barrett *et al*.^58^ were not significant, showing poor capability for both D_K_ and K at separating low- and high-grade lesions. Correlation analysis for the DKI parameters, evaluating their relationship with GS, revealed a significatively positive correlation between K and GS^48,52,53,55^, and a significantly negative correlation between D_K_ and GS^48,52^. Suo *et al*.^55^, however, found no significant correlation between D_K_ value and GS.

### Results of meta-analyses

In order to perform the meta-analyses indicated in Fig. 2, 17 studies were fully excluded from previous selection. Data from the same study were considered multiple times in the following cases: different b-value ranges were used to perform acquisition/processing of DW-MRI images; PCa tissue was compared with different zones of NT (e.g. PCa vs normal TZ and PCa vs normal PZ); NT was compared with different PCa zones (e.g. NT vs PCa in TZ and NT vs PCa in PZ); NT was compared with PCa with different GS (e.g. NT vs PCa with GS = 6 and NT vs PCa with GS ≥ 7); low grade PCa was compared to more than one PCa with higher GS. Data from the study of Langkilde *et al*.^15^ were extracted only for the analyses including b = 0 s/mm^2^.

Finally, as for PCa detection, studies concerning DKI and SE were respectively 32, and 10. For the SE, 1770 regions (694 positive) were analyzed; for the DKI, 5167 regions (2136 positive) were analyzed for K and 3331 (1439 positive) for D_K_.

As for characterization, the meta-analysis was performed only for DKI model, because there were no sufficient SE data available. DKI was addressed by 9 studies, with 897 lesions (300 low-grade) included for K and 440 (154 low-grade) for D_K_.

The overall quality of included studies in meta-analyses, for both PCa detection and characterization, was considered good for our purposes (See Supplementary Materials –S4-).

Concerning IVIM and BE models, the high heterogeneity in applied fitting functions and procedures (see Table 2) led us to decide not to perform any quantitative analysis on these models.

### Performance of models in PCa detection

**Figure 4:**
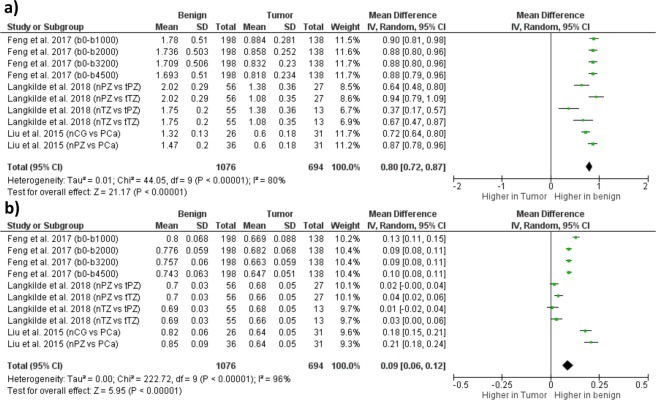
Forest plot showing results on Stretched Exponential Model (SE) parameters for PCa detection: (**a**) results on distributed diffusion coefficient (DDC) in normal tissue (NT) PCa [mean DDC ± standard deviation (SD) × 10^−3^ mm^2^/s]; (**b**) results on heterogeneity index (α) in NT and PCa [mean α ± SD]. Studies included in the meta-analysis are listed in the column “Study or Subgroup” and, in case of studies considered multiple times, the different b-value ranges and/or outcome measurements are reported in parenthesis. Abbreviations: nPZ, normal peripheral zone; tPZ, tumoral peripheral zone; nTZ, normal transitional zone; tTZ, tumoral transitional zone; nCG, normal central gland.

**Figure 5:**
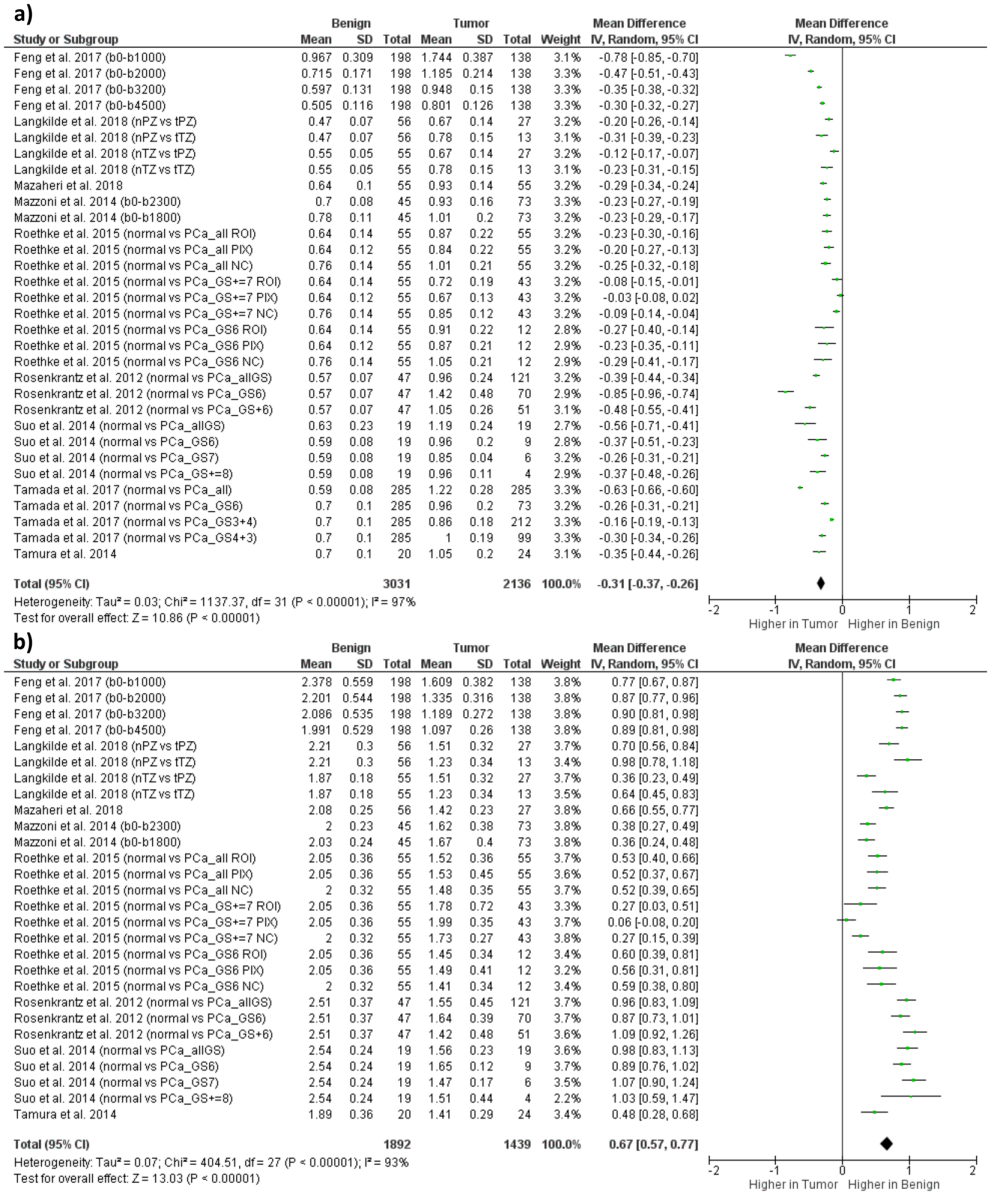
Forest plot showing results on Diffusion Kurtosis Imaging (DKI) model parameters for prostate cancer (PCa) detection: (**a**) results on kurtosis coefficient (K) in normal tissue (NT) and PCa [mean ± standard deviation (SD) × 10^−3^ mm^2^/s]; results on diffusion coefficient corrected for kurtosis (D_K_) in NT and PCa [mean ± SD × 10^−3^ mm^2^/s]. Studies included in the meta-analysis are listed in the column “Study or Subgroup” and, in case of studies considered multiple times, the different b-value ranges and/or outcome measurements are reported in parenthesis. Abbreviations: nPZ, normal peripheral zone; tPZ, tumoral peripheral zone; nTZ, normal transitional zone; tTZ, tumoral transitional zone; PCa_all, all PCa lesions; PCa_GS, PCa lesions with Gleason Score equal to a certain value; PCa_GS+, PCa lesions with Gleason Score greater to a certain value; PCa_GS+ = , PCa lesions with Gleason Score greater or equal to a certain value; ROI, region of interest (ROI)-based fitting approach; PIX, voxel-by-voxel fitting approach; NC, ROI-based fitting approach without noise correction.

The random-effect analysis was used for all SE and DKI parameters. The heterogeneity was high (I^2^ > 50% and P < 0.0001). D_K_ was significantly higher in NT, while K was significantly higher in PCa tissue. These results are highlighted by the forest plot in Figs 4 and 5.

### Performance of models in PCa characterization

**Figure 6:**
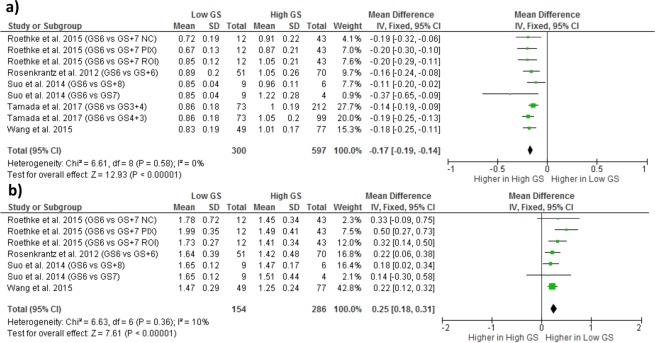
Forest plot showing results on DKI model parameters for prostate cancer (PCa) characterization: (**a**) results on diffusion kurtosis (K) in low and high Gleason Score (GS) PCa [mean ± standard deviation (SD) × 10^−3^ mm^2^/s]; (**b**) results on diffusion coefficient corrected for kurtosis (D_K_) in low and high GS PCa [mean ± SD × 10^−3^ mm^2^/s]. Abbreviations: GS, PCa lesions with Gleason Score equal to a certain value; PCa_GS+, PCa lesions with Gleason Score greater to a certain value; ROI, region of interest (ROI)-based fitting approach; PIX, voxel-by-voxel fitting approach; NC, ROI-based fitting approach without noise correction.

D_K_ was found to be significantly higher in PCa lesions with low GS, while K was significantly higher in those with high GS. These results are highlighted by the forest plot in Fig. 6.

### Publication bias

Since the number of included studies was superior to 10 only in meta-analyses based on detection and involving DKI parameters (D_K_ and K), publication bias was evaluated plotting funnel plots only in these cases. As shown in Supplementary Materials –S5-, all plots suggested a low risk of publication bias.

## Discussions

In this study we investigated the potential value of the most commonly used Non-Gaussian DWI models for detection and characterization of prostate cancer. ME DWI has been shown to be a useful tool for PCa diagnosis, not only for detection of prostatic lesions, but also for characterization of disease aggressiveness^8,9,16,17^. However, in the last years, due to the limited assumption at the basis of this approach, many Non-Gaussian diffusion models have been proposed to better depict diffusion signal^19,20,23,24^ and more and more studies investigated their diagnostic role and clinical value on PCa^13,15,31,33–50,52–61^. Due to the large amount of studies and data contained therein, and the not always clear results regarding the benefits of using Non-Gaussian DWI for PCa diagnosis, we performed a systematic review followed by a meta-analysis, reviewing their parameters and trying to understand if they could provide additional information. We initially examined all found studies concerning both Non-Gaussian and Gaussian models for detection and characterization of prostate cancer from 2012 onwards. This qualitative analysis involved 29 studies: results and conclusions of selected studies varied from each other, often showing inconsistence and not a clear idea about the actual usefulness and the added value that Non-Gaussian model parameters may led to standard DWI.

In particular, among IVIM parameters, D seemed to be the most useful for PCa diagnosis, while there was no clarity on the effective significance and usefulness of the parameters related to perfusion, D* and f. DKI parameters, K and D_K_, according to a large number of examined studies were considered to be useful for PCa detection, but a clear evidence about DKI better performance had not still be reached.

Compared to those on IVIM and DKI, a smaller quantity of studies on BE and SE was present in literature, so it was even more complicated to assess the ability of these models to help in PCa detection and characterization. Among SE parameters, only DDC seemed to show significance, while conflicting views were expressed on α. About BE, there were conflicting or not significant results for D_slow_, D_fast_, f_fast_, and also a not clear interpretation of their physiological basis^45^.

In this scenario, it would be desirable to reach a common view on the use of Non-Gaussian DWI models in addiction or in substitution of the standard DWI protocol for PCa diagnosis. With our systematic review, we summarized the available data reported in previously selected studies, and, if possible, for each Non-Gaussian model, we quantitatively evaluated the capability to differentiate between NT and PCa and between low- and intermediate/high-GS lesions. The high heterogeneity in applied functions and fitting procedures due to a not yet existing consensus on the best processing approach to fit biexponential models^62^ led us to not perform any statistical analysis on IVIM and BE models. Moreover, the small quantity of studies on SE models did not allow us to conduct statistical analysis on the power of α and DDC to classify lesions on the basis of GS.

Our results showed that, concerning PCa detection, K, D_K_, DDC and α showed statistical significance, allowing to distinguish prostate cancer from NT. Lower values of D_K_ and DDC found in PCa could be linked to the destruction of the prostatic structure-like acini together with a higher cellular and stroma density typical of PCa tissues^49,55^. The significantly higher value of K in PCa could be associated with the increased microstructural complexity of prostate cancer^54,55^. On the contrary, α was found to be significantly lower in PCa and this is in accordance to its association with histological heterogeneity, clearly present in prostate cancer tissue^49^. It should be considered that studies involving SE were fewer than those selected for DKI and so, although the results on DDC and α showed statistical significance, more studies would be required to validate this result. Given the high number of included studies, and the significative performance of both its parameters, DKI model claimed to be helpful for the detection of PCa.

Among outcomes regarding characterization of PCa aggressiveness, only K and D_K_, showed statistical significance, differentiating lesions according to GS. The significantly greater value of D_K_ in intermediate/high-lesions highlighted reduced diffusion of water in these PCa than in those characterized by a GS equal or lower than 6, and it could be linked to the progressive increased cellularity of malignant lesions^54^. The significantly greater value of Kurtosis (K) in intermediate/high-lesions highlight reduced diffusion of water in these lesions, and it could be linked to the progressive increased microstructural complexity of malignant lesions^52–55^. The computed heterogeneities were lower than the ones found in the analyses on PCa detection, but it is not so reliable due to the lower number of studies involved^63^.

The obtained high heterogeneities, both in PCa detection and characterization, may be caused by several factors that match to the limitations of the study, which are highlighted in the following subparagraph.

### Limitations of the study

This meta-review suffers from several limitations which deserve to be discussed.

As concern patient characteristics, we did not consider patient age and did not draw distinction between different kind of NT ROIs (e.g. placed on prostate of healthy volunteers or on healthy prostate zones of PCa patients) or between the different examined anatomic zone (TZ, PZ, CG). Also, we have not considered the different pathologic reference standard used across studies, be it biopsy or radical prostatectomy specimens, which may lead to assign different Gleason scores for the same prostatic lesion^64,65^.

As to characteristics of DWI protocol, because of the lack of a standard b-value range for PCa, b-values used to acquire DWI signal differed across selected studies, probably contributing to increase heterogeneity due to the supposed dependence of the computed parameters on the adopted b-values^34,36^. Specifically for IVIM parameters, the high variability found across studies can also be explained by the effect of a non-constant Echo Time (TE)^66,67^ and of a variable number of selected b-values^68^.

Moreover, the different applied diffusion time, which was found to be related to ADC and fractional anisotropy (FA) by Bourne *et al*.^69^ could have influenced results, but only four of selected studies reported information on diffusion time parameters^15,35,44,47^ (see Supplementary Material –S6- for more details). Further studies aimed at finding a standard DW-MRI protocol for PCa are required.

It could be also interesting to explore the huge sources of heterogeneity and their effects on parameters performances in PCa diagnosis using a meta-regression and subgroup analyses, in order to perform a diagnostic test accuracy (DTA) meta-analysis and obtain quantitative comparison of each Non-Gaussian models with mono-exponential model, as done, for example, by Si *et al*.^70^ for DKI. However, selected studies, especially those covering PCa characterization and BE or SE, did not reported sufficient data to build contingency tables, calculate quantitative measures and construct summary ROC curves. Moreover, too few studies on PCa characterization provided correlation coefficient values between parameters and GS to perform a correlation meta-analysis.

With respect to data analysis characteristics, we have not made any differences between 2D and 3D ROIs. In addition, we included studies that used both ROI-based parameters extraction approach (*i.e*., fitting procedure over the mean signal intensity values in previously traced ROIs) and voxel-based approach (*i.e*., voxel-by-voxel fitting procedure and subsequent ROI measurement on the resulting parametric map^59^). Further, there was neither an established fitting procedure to obtain parameters belonging to the same Non-Gaussian model and this made it impossible to perform any statistical analysis on certain models such as IVIM and BE. Larger studies are required to find a standard fitting function/procedure.

Moreover, it is not to be neglected the lack of access to data in the selected studies, which prevents the external validation of obtained results by independent researchers^71^. Only Feng *et al*.^36^ took a step toward data sharing, providing, in the Supporting Material of their work, a full table including the estimated DWI parameters for each patient included in the study.

Data availability issue is gaining an increasing importance in research and in particular in MR community^72^. Some goals of data sharing should be the creation of reference findings/algorithms/implementations that can be used for comparison when publishing new methods or the possibility to compare individual results, for example in terms of consistency, computation time and hardware/programming language requirements. To our knowledge, among research teams dealing with prostate cancer diagnosis, only the groups of Chaddad *et al*.^73^ and Jambor *et al*.^74^ make their data fully available. With respect to our meta-analysis, the access to external data would have allowed not only to go beyond the traditional meta-analysis of intervention venturing into more complex meta-analytic approaches^75^, but it would have also helped to shed light in the issue of standardization of DWI protocols and fitting functions/procedures for Non-Gaussian DWI parameters. The absence of standardization directly affects repeatability and reproducibility for DWI parameters that are fundamental to allow a fair and robust comparison among studies, leading to a more powerful meta-analysis^71^. In addition, repeatability and reproducibility are also essential in clinical practice for a correct treatment planning, response monitoring and to use DWI as clinical biomarker for cancer diagnosis^76–82^. To our knowledge, there are only few studies on Non-Gaussian DWI models for PCa diagnosis approaching these issues. Merisaari *et al*.^44^ evaluated five different IVIM fitting methods using a population of 81 patients who underwent DWI examination twice, revealing poor reproducibility of IVIM parameters. Jambor *et al*.^46^ addressed for repeatability of ADC, SE, DKI and BE parameters involving a smaller patient population of 24 patients who underwent four DWI examinations, showing low repeatability for BE fitted parameters and a higher and similar repeatability among ADC, SE and DKI fitted parameters. To our knowledge, current literature lacks of studies concerning reproducibility of Non-Gaussian DWI parameters for PCa diagnosis across different centers, scanners, and readers. This is a direct consequence of the above-mentioned lack of standardization in DWI protocol, fitting functions/procedures, and also automatic tools for ROI placement^44^.

Nevertheless, the strength of this work was that we assessed not only tumor detection, as in other reviews^70^, but also PCa characterization in terms of aggressiveness. In addition, multiple Non-Gaussian models were considered.

## Conclusions

The role of Non-Gaussian diffusion models in the detection and characterization of PCa aggressiveness and, consequently, in making clinical decisions, remains questionable. In this context, this article, highlighting the challenges that have emerged when comparing studies between each other, may serve as a starting point for future studies evaluating Non-Gaussian DWI performances to give a more precise biophysical interpretation of their parameters with the objective of identifying a standardized DW-MRI protocol in PCa diagnosis.

## Supplementary information

Supplementary Materials
