# Supplementary material for: Non-Gaussian models of diffusion weighted imaging for detection and characterization of prostate cancer: a systematic review and meta-analysis
Source: Sci Rep. 2019 Nov 14;9:16837. doi: 10.1038/s41598-019-53350-8 (PMC6856159; doi:10.1038/s41598-019-53350-8)
Supplement: Supplementary file 1 — Supplementary Materials [file 41598_2019_53350_MOESM1_ESM.pdf]

Non-Gaussian models of diffusion weighted imaging for detection and characterization of prostate cancer: a systematic review and meta-analysis

Brancato V. [1], Cavaliere C. [1,*], Salvatore M. [1], Monti S. [1]

[1] IRCCS SDN, Napoli
[*] corresponding author

S1. Diffusion Weighted MRI Mathematical Models in PCa

Gaussian Model

The simplest mathematical model developed to evaluate DWI is the mono-exponential model, which presumes that the water molecules move freely and the probability function of their displacement follows a Gaussian distribution[18]. This model is described by the equation:

$$\frac{S(b)}{S_0} = exp\,(-b \cdot ADC)$$

where *S(b)* is the measured signal intensity at a certain b, S_0 is the signal intensity without the influence of diffusion, b is a factor that measures the degree of diffusion weighting applied, and *ADC* is the Apparent Diffusion Coefficient, an average value related to diffusion.

Non-Gaussian Models

Intravoxel Incoherent Motion Model (IVIM)

The IVIM model, as described by Le Bihan et al.[19] in 1988, is a bicompartimental model linking the greater attenuation of diffusion signal occurring at low b-values (b<200 mm^2/s) to microcirculation of blood in the capillary network (perfusion). Diffusion and perfusion contributions can be separated using the biexponential decay function:

$$\frac{S(b)}{S_0} = (1-f) \cdot exp(-b \cdot D) + f \cdot exp\,(-b \cdot (D^* + D))$$

where f is the perfusion fraction (vascular volume fraction), D the molecular diffusion coefficient and D* the so-called pseudo-diffusion coefficient, related to blood velocity and mean capillary segment length, which becomes insignificant for high b-values (>200 mm2/s).

Biexponential model (BE)

When b-values over than 1000 mm^2/s are used and low b-values are not acquired, the two components of biexponential diffusion decay signal cannot be explained by IVIM model. So, a biexponential model for high b-values was introduced by Mulkern et al.[20,21]and is represented by following equation:

$$\frac{S(b)}{S_0} = f_{slow} \cdot exp(-b \cdot D_{slow}) + f_{fast} \cdot exp\,(-b \cdot D_{fast})$$

where D_{fast} and D_{slow} are fast and slow diffusion coefficients, respectively, and f_{fast} and f_{slow} their amplitudes. Le Bihan[22] suggested that D_{slow} may represent water linked with cell membranes and cytoskeleton structures (restricted diffusion), while D_{fast} the less restricted water found in intra- and extra-cellular space, characterized by a Gaussian unrestricted and slightly hindered diffusion.

Stretched exponential Model (SE)

The SE model was introduced by Bennet et al.[23]to simply consider the deviation from mono-exponential trend using the following two-parameter stretched exponential equation:

$$\frac{S(b)}{S_0} = exp\,[-(b \cdot DDC)^{\alpha}]$$

where α is the so-called heterogeneity index, describing the deviation from a single exponential decay, and DDC is the distributed diffusion coefficient which can be considered as a weighted sum over a distribution of ADCs that comprises the multi-exponential decay properties. The diffusion signal intensity decay is therefore described as a continuous distribution of sources decaying at different rates.

Diffusion Kurtosis Model (DKI)

The DKI model, first described by Jensen et al.[24]in 2005, quantifies the deviation of tissue diffusion from a Gaussian behavior due to diffusion barriers, such as cell membranes and organelles or other hindrance due to complex and restricted structures in tissues. This model considered the microstructural complexity of tissues better than standard DWI. The relation between the diffusion signal intensity and the b-factors can be expressed with the following equation:

$$\frac{S(b)}{S_0} = exp\,(-b \cdot D + \frac{b^2 \cdot {D_K}^2 \cdot K}{6})$$

where D_K is an analog of ADC corrected for non-Gaussian behavior and K is the kurtosis coefficient expressing the deviation from gaussianity.

S2. Key terms used in literature search

- “prostate cancer and non-gaussian diffusion MRI”
- “prostate cancer and comparison of gaussian and non-gaussian diffusion MRI”
- “prostate cancer and IVIM”
- “prostate cancer and intravoxel incoherent motion MR imaging”
- “prostate cancer and biexponential diffusion model”
- “prostate cancer and stretched exponential diffusion MRI”
- “prostate cancer and DKI diffusion”
- “prostate cancer and diffusion kurtosis imaging”
- “prostate cancer detection and IVIM diffusion MRI”
- “prostate cancer detection and biexponential diffusion MRI”
- “prostate cancer detection and stretched exponential diffusion MRI”
- “prostate cancer detection and diffusion kurtosis MRI”
- “prostate cancer and characterization of aggressiveness and non-gaussian diffusion”
- “prostate cancer and characterization of aggressiveness and IVIM”
- “prostate cancer and characterization of aggressiveness and biexponential and diffusion”
- “prostate cancer and characterization of aggressiveness and stretched exponential and diffusion”
- “prostate cancer and characterization of aggressiveness and DKI”
- “prostate cancer and gleason score and non-gaussian diffusion”
- “prostate cancer and gleason score and IVIM”
- “prostate cancer and gleason score and biexponential and diffusion”
- “prostate cancer and gleason score and stretched exponential and diffusion”
- “prostate cancer and gleason score and DKI

S3. PRISMA Checklist

Section/topic	#	Checklist item	Reported on page #
**TITLE**			
Title	1	Identify the report as a systematic review, meta-analysis, or both.	1
**ABSTRACT**			
Structured summary	2	Provide a structured summary including, as applicable: background; objectives; data sources; study eligibility criteria, participants, and interventions; study appraisal and synthesis methods; results; limitations; conclusions and implications of key findings; systematic review registration number.	1
**INTRODUCTION**			
Rationale	3	Describe the rationale for the review in the context of what is already known.	1-2
Objectives	4	Provide an explicit statement of questions being addressed with reference to participants, interventions, comparisons, outcomes, and study design (PICOS).	2
**METHODS**			
Protocol and registration	5	Indicate if a review protocol exists, if and where it can be accessed (e.g., Web address), and, if available, provide registration information including registration number.	NA
Eligibility criteria	6	Specify study characteristics (e.g., PICOS, length of follow-up) and report characteristics (e.g., years considered, language, publication status) used as criteria for eligibility, giving rationale.	3
Information sources	7	Describe all information sources (e.g., databases with dates of coverage, contact with study authors to identify additional studies) in the search and date last searched.	3
Search	8	Present full electronic search strategy for at least one database, including any limits used, such that it could be repeated.	3, S2
Study selection	9	State the process for selecting studies (i.e., screening, eligibility, included in systematic review, and, if applicable, included in the meta-analysis).	5
Data collection process	10	Describe method of data extraction from reports (e.g., piloted forms, independently, in duplicate) and any processes for obtaining and confirming data from investigators.	5
Data items	11	List and define all variables for which data were sought (e.g., PICOS, funding sources) and any assumptions and simplifications made.	5

Risk of bias in individual studies	12	Describe methods used for assessing risk of bias of individual studies (including specification of whether this was done at the study or outcome level), and how this information is to be used in any data synthesis.	4-5
Summary measures	13	State the principal summary measures (e.g., risk ratio, difference in means).	5
Synthesis of results	14	Describe the methods of handling data and combining results of studies, if done, including measures of consistency (e.g., I^2) for each meta-analysis.	5

**Section/topic**	**#**	**Checklist item**	**Reported on page #**
Risk of bias across studies	15	Specify any assessment of risk of bias that may affect the cumulative evidence (e.g., publication bias, selective reporting within studies).	5
Additional analyses	16	Describe methods of additional analyses (e.g., sensitivity or subgroup analyses, meta-regression), if done, indicating which were pre-specified.	NA
**RESULTS**			
Study selection	17	Give numbers of studies screened, assessed for eligibility, and included in the review, with reasons for exclusions at each stage, ideally with a flow diagram.	5, 11
Study characteristics	18	For each study, present characteristics for which data were extracted (e.g., study size, PICOS, follow-up period) and provide the citations.	6-8
Risk of bias within studies	19	Present data on risk of bias of each study and, if available, any outcome level assessment (see item 12).	11, S4
Results of individual studies	20	For all outcomes considered (benefits or harms), present, for each study: (a) simple summary data for each intervention group (b) effect estimates and confidence intervals, ideally with a forest plot.	12-15
Synthesis of results	21	Present results of each meta-analysis done, including confidence intervals and measures of consistency.	12-15
Risk of bias across studies	22	Present results of any assessment of risk of bias across studies (see Item 15).	15, S5
Additional analysis	23	Give results of additional analyses, if done (e.g., sensitivity or subgroup analyses, meta-regression [see Item 16]).	NA
**DISCUSSION**			

Summary of evidence	24	Summarize the main findings including the strength of evidence for each main outcome; consider their relevance to key groups (e.g., healthcare providers, users, and policy makers).	15-17
Limitations	25	Discuss limitations at study and outcome level (e.g., risk of bias), and at review-level (e.g., incomplete retrieval of identified research, reporting bias).	16-17
Conclusions	26	Provide a general interpretation of the results in the context of other evidence, and implications for future research.	17
**FUNDING**			
Funding	27	Describe sources of funding for the systematic review and other support (e.g., supply of data); role of funders for the systematic review.	NA

From: Moher D, Liberati A, Tetzlaff J, Altman DG, The PRISMA Group (2009). Preferred Reporting Items for Systematic Reviews and Meta-Analyses: The PRISMA Statement. PLoS Med 6(7): e1000097. doi:10.1371/journal.pmed1000097

For more information, visit:**www.prisma-statement.org**.

S4. Quality assessment results

Meta-analyses on PCa detection

Stretched Exponential Model (SE)

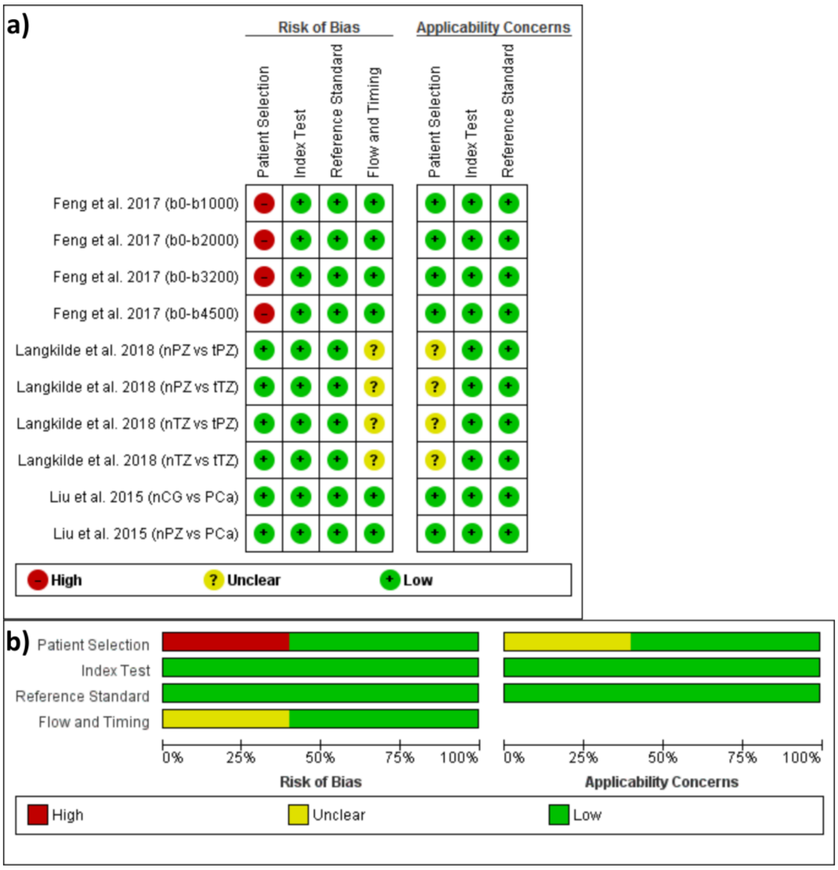

Figure S4.1: Quality assessment for meta-analysis on Stretched Exponential model (SE) capability of detecting prostate cancer (PCa). (a) Risk of bias summary. (b) Risk of bias graph.

Diffusion Kurtosis Imaging (DKI)

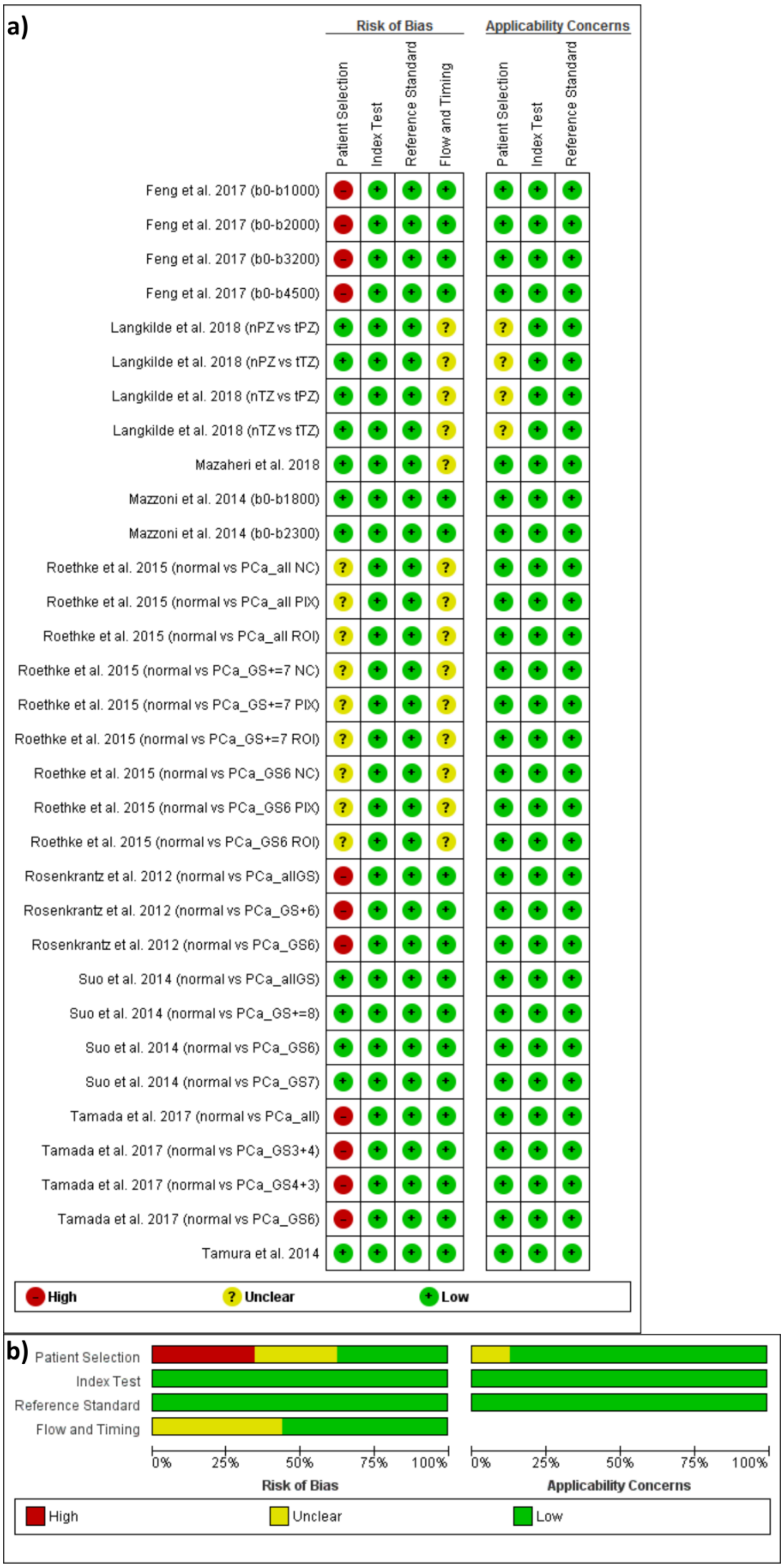

Figure S4.2: Quality assessment for meta-analysis on Diffusion Kurtosis Imaging (DKI) capability of detecting prostate cancer (PCa). (a) Risk of bias summary. (b) Risk of bias graph. Abbreviations: nPZ, normal peripheral zone; tPZ, tumoral peripheral zone; nTZ, normal transitional zone; tTZ, tumoral transitional zone; PCa_all, all PCa lesions; PCa_GS, PCa lesions with Gleason Score equal to a certain value; PCa_GS+, PCa lesions with Gleason Score greater to a certain value; PCa_GS+=, PCa lesions with Gleason Score greater or equal to a certain value; ROI, region of interest (ROI)-based fitting approach; PIX, voxel-by-voxel fitting approach; NC, ROI-based fitting approach without noise correction.

Meta-analyses on PCa characterization

Diffusion Kurtosis Imaging (DKI)

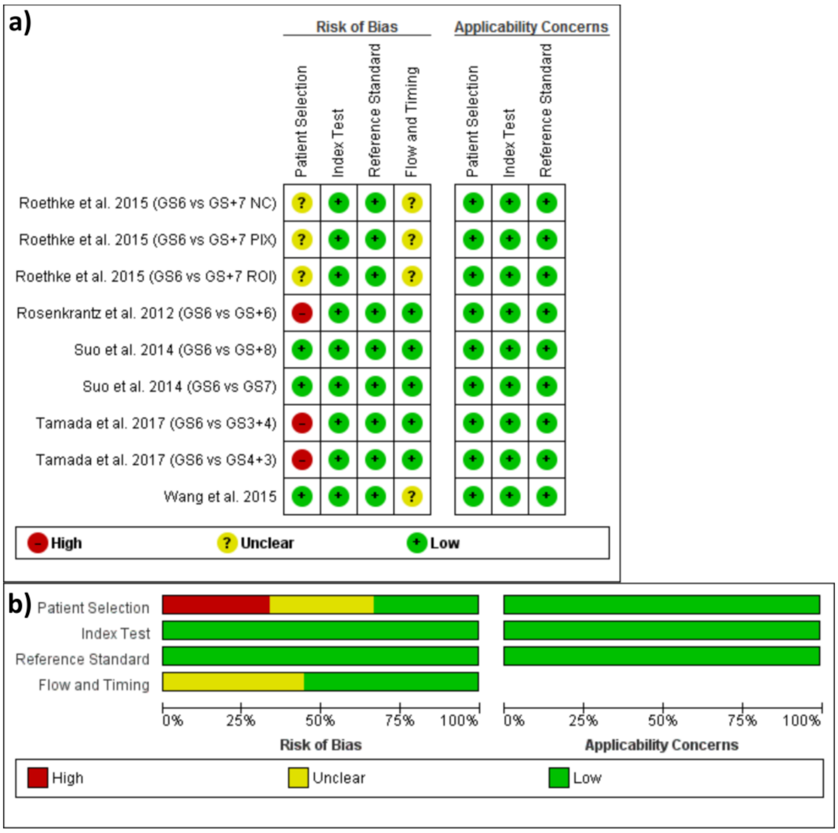

Figure S4.3: Quality assessment for meta-analysis on Diffusion Kurtosis Imaging (DKI) capability of distinguishing low- from high-grade prostate cancer (PCa). (a) Risk of bias summary. (b) Risk of bias graph. Abbreviations: GS, PCa lesions with Gleason Score equal to a certain value; PCa_GS+, PCa lesions with Gleason Score greater to a certain value; ROI, region of interest (ROI)-based fitting approach; PIX, voxel-by-voxel fitting approach; NC, ROI-based fitting approach without noise correction.

S5. Funnel plots inspected to detect publication bias

Diffusion Kurtosis Imaging (DKI)

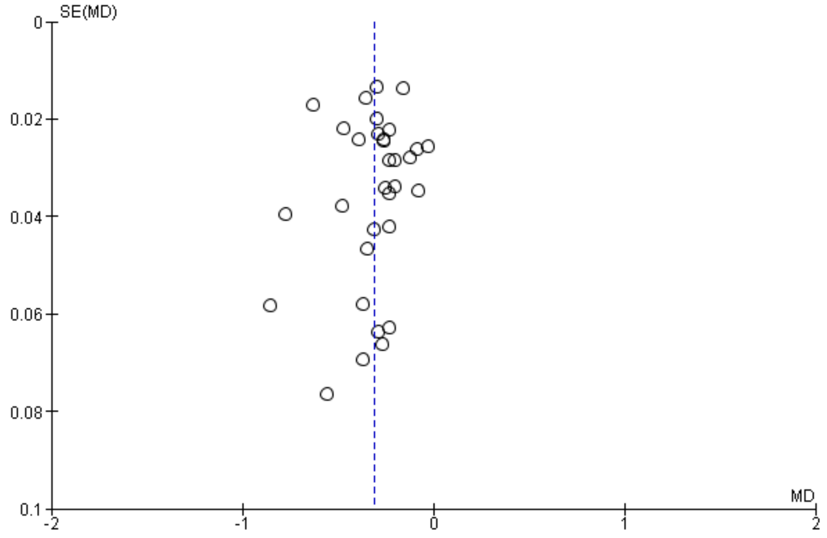

Figure S5.1: funnel plot for Kurtosis (K) of Diffusion Kurtosis Imaging (DKI) model in meta-analysis for prostate cancer (PCa) detection. Abbreviations: MD, Mean Difference; SE, Standard Error.

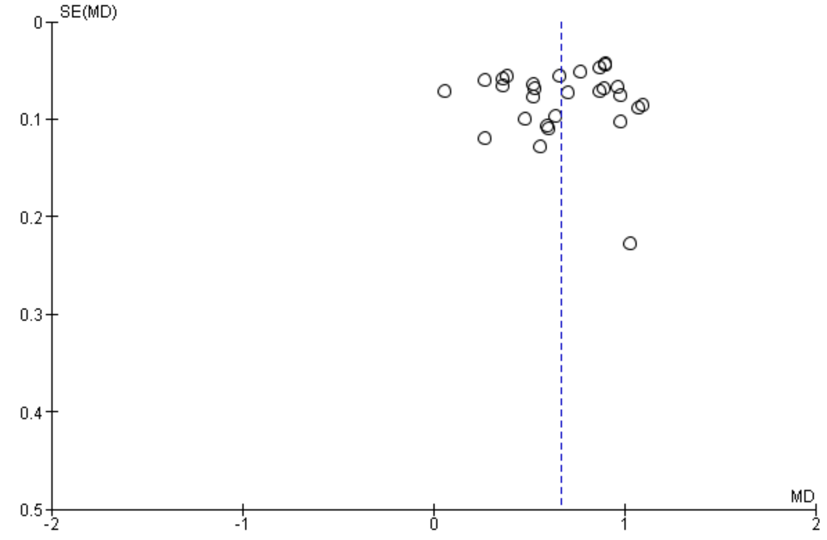

Figure S5.2: funnel plot for diffusion coefficient corrected for kurtosis (D_K) of Diffusion Kurtosis Imaging (DKI) model in meta-analysis for prostate cancer (PCa) detection. Abbreviations: MD, Mean Difference; SE, Standard Error.

S6. Diffusion times

Among the 29 studies included in the systematic review, only 4 of these provided information on diffusion times: Ueda et al. (2015); Merisaari et al. (2017); Mazaheri et al. (2018); Langkilde et al. (2018).
We provide them below:

Author	δ [ms]	Δ [ms]	DT [ms]
Ueda et al.	19.2	33.2	26.8
Merisaari et al.	6.60	19.004	16.804
Mazaheri et al.	NR	NR	24.5
Langkilde et al.	37	47	35

Table S6.1 Information on diffusion times. Abbreviations: δ (delta) = diffusion gradient duration; Δ (DELTA) = diffusion gradient timing; DT = diffusion time (Δ-δ/3); NR = Not Reported.
